# “From my life, she will never be gone, even though she is no longer here” – a single case interpretative phenomenological analysis on spousal loss and resilience

**DOI:** 10.1080/17482631.2025.2477372

**Published:** 2025-03-13

**Authors:** Chloe Beeris, Alistair Niemeijer, Rob Bisseling, Anja Machielse

**Affiliations:** a Humanism and Social Resilience, University of Humanistic Studies (UvH), Utrecht, The Netherlands; b Care Ethics and Policy, University of Humanistic Studies (UvH), Utrecht, The Netherlands; c Independent Scholar, Stichting Erwelzijn, Deventer

**Keywords:** Ageing, case study, interpretative phenomenological analysis, Resilience, spousal loss

## Abstract

Older people often face drastic life events, such as spousal loss, that profoundly affect their daily lives. Consequently, resilience—how one navigates life’s changes to avoid further adverse outcomes—is increasingly relevant in ageing studies. Although understanding older adults’ resilience is key to preventing adverse outcomes, the complexity of loss-related events and everyday resilience in later life is underexplored from a process-based perspective. This study employs an Interpretative Phenomenological Analysis (IPA) case study of one individual to investigate resilience in response to spousal loss from a process-based perspective. Four interviews were conducted with this one participant and data was analysed following IPA guidelines. Findings indicate how resilience, in this case, resembles a process of continuous adaptation and renewal or “bouncing forward”, in the face of diverse adversities, as written by Bourbeau. This study enriches our understanding of the process-based perspective on resilience, which is essential for concretely defining resilience and its practical application.

## Introduction

Spousal loss is recognized by several authors as one of the most stressful life events to occur in one’s life (Holmes & Rahe, 1967; Infurna & Luthar, 2017; Yu et al., 2021). Such loss is prevalent in later life and often entails shattering of a “taken for granted” world (Fang & Carr, 2024). This requires (re)defining meaning of identity and existence after losing a relationship that has likely influenced everyday life and identity (Fang, 2022; Fang & Carr, 2024; Larsson et al., 2017; Naef et al., 2013; Richardson, 2013). This major life event is well documented as having several potential injurious outcomes (Stahl & Schulz, 2013) such as intense suffering (Holmes & Rahe, 1967), decline in physical and mental health (Ang, 2023; Buckley et al., 2012; Jones et al., 2010; Lee & Carr, 2007; Shear et al., 2013; Utz et al., 2012) and higher risk of mortality (Holland et al., 2013; Manzoli et al., 2007; van den Berg et al., 2011). Why injurious outcomes occur and persist in later life for some people but not for others remains a question of wide interest (Beeris et al., 2022). The concept of resilience is valuable in this regard. It illustrates how people navigate adversities to prevent further adverse outcomes (Amaral et al., 2021; Browne-Yung et al., 2017; Hayman et al., 2017; Wiles et al., 2012; Wild et al., 2011; Wiles et al., 2019; Wilson et al., 2020).

In research on resilience in later life, emphasis is often placed on resource- and outcome-based perspectives on resilience and a detailed, concrete understanding of resilience from a process-based perspective is lacking (Beeris et al., 2022; Glas et al., 2023). Put simply, the helpful components and objectives for resilience in later life are clear; however, understanding of the mechanisms involved in applying them in real-life settings, as well as how one experiences this, remains underdeveloped. Gaining deeper insight into these mechanisms is crucial for accurately defining resilience and informing how resilience operates and is experienced in practice. This knowledge informs appropriate and timely resilience-enhancing practices, potentially preventing further adverse outcomes when navigating life’s challenges.

In light of these considerations, this paper aims to develop an understanding of the mechanisms that facilitate different outcomes when navigating adversity and how these mechanisms are experienced. To achieve this, an IPA case study of one individual is utilized to examine the interaction of adversity, outcomes, and the underlying mechanisms involved in navigating the highly disruptive and prevalent life event of spousal loss. Specifically, this paper seeks to answer the following research question: How does one experience navigating the adversities associated with spousal loss to prevent the occurrence and persistence of further injurious outcomes?

## Theoretical framework

### Spousal loss

In this paper, we identify spousal loss to involve two distinct phenomena: bereavement and widowhood. According to Bennett and Soulsby (2012) on one hand, bereavement regards the state of having experienced the death of one’s spouse and the personal consequences and meanings this brings. Widowhood on the other hand, is a long-term state that does not only carry personal consequences but also carries long-term social consequences and meanings such as changes in friendships, social support, and changes in status within society. One can experience high levels of resilience in bereavement for example, and struggle with doing so in widowhood (Bennett, 2010).

The grieving process experienced during spousal loss is also widely written about. Freud (1957) largely influenced early theories on grief with his paper on *Mourning and Melancholia* (Ang, 2023; Hall, 2014). He recommended for example the severing of energetic ties with the deceased in order to be able to move forward. Following Freud, stage theories became popular, structuring grief into a series of stages and tasks (Ang, 2023; Hall, 2014), the most well-known being from Kübler-Ross (1969). Several authors, however, emphasize that while stage theories offer valuable insights, they fail to fully capture the complexity and idiosyncratic nature of the grieving experience (Ang, 2023; Hall, 2014; Stroebe & Schut, 2010; Worden, 2008). Furthermore, Klass et al. (1996) emphasize the concept of “continued bonds,” asserting that death does not signify the end of a relationship. They highlight a potentially beneficial role of maintaining these bonds, even though the other has passed away. Two of the most widely influential contemporary models of grief seek to capture the complexity of both the grieving process and continued bonds (Hall, 2014), namely the Dual Process Model of Grief (Stroebe & Schut, 2010) and the Task-Based Model (Worden, 2008).

### Resilience

Resilience is challenging to define and lacks a standardized definition (Angevaare et al., 2020). Nonetheless, consensus is strengthening that an operable definition of resilience should incorporate three elements: adversity, outcome, and mechanisms (Angevaare et al., 2020; IJntema et al., 2023; Van Breda, 2018). Firstly, adversity can be defined as “particular (often extraordinary) embodied and emplaced circumstances in people’s lives that cause pain, disruption, exhaustion, disorientation, loneliness, and grief” (Power et al., 2019, p. 2). Adversity is also referred to in literature on resilience in later life as a stressor, challenge, risk, unfavourable condition or threat (Angevaare et al., 2020). Secondly, outcome refers to developments that unfold as a consequence of facing adversity. See for example, the psychological immunity–psychological elasticity (PI-PE) model which illustrates adaption to adversity through thriving, sustainability, recovery or transformation, or maladaptation through rigidity and vulnerability (IJntema et al., 2023). Finally, the third essential element, mechanisms, are the interactions that facilitate varying outcomes when navigating adversity (Angevaare et al., 2020). These interactions occur within and between several levels, that is, individual, community and society and incorporate engagement of essential factors or resources.

Different perspectives can be utilized to deepen understanding of how resilience works when facing challenging life events, such as spousal loss; namely, resource-based, outcome-based, and process-based perspectives (Beeris et al., 2022). Firstly, resource-based perspectives focus on the essential factors or resources of individuals or groups identified as being “resilient”, for example physical, mental and cognitive health, living conditions and social support (Angevaare et al., 2020). These resources and factors are identified across multiple levels—individual, community, and societal—demonstrating that resilience extends beyond personal traits or capacities to include broader contextual and relational dimensions. Secondly, outcome-based perspectives focus on the outcomes that unfold when an individual or a group of people is faced with adversity. What authors consider to be a “resilient outcome” however varies significantly (Angevaare et al., 2020; Beeris et al., 2022). As highlighted by Luthar and Brown (2007) resilience is never an “across the board” phenomenon. There are a range of outcomes, from more negative to more positive (Condly, 2006). Thirdly, process-based perspectives focus on the mechanisms involved in navigating adversity and facilitating varying outcomes, emphasizing that these mechanisms manifest dynamically depending on timing and context throughout one’s life course (Beeris et al., 2022; Glas et al., 2023; Voie et al., 2024). See, for example, how Bourbeau (2018) describes resilience as a process of “bouncing forward.” This process of “bouncing forward” conceptualizes how individuals navigate the dynamic, ever-shifting, and context-dependent interaction of adversity and outcomes across one’s lifespan. Rather than “bouncing back” to a pre-adversity state or progressing towards static and fixed outcomes, this interaction fosters continuous adaptation and renewal.

## Research design and methods

### Research design

This paper employs a case study of one individual in combination with Interpretative Phenomenological Analysis (IPA) which is a technique endorsed by Smith et al. (2022). This is, nevertheless, quite distinctive as “the balance between case studies and studies using larger sample sizes is currently biased in favour of the latter, to the extent that it disadvantages case studies in most disciplines” (Flyvbjerg, 2006, p. 242). Single-case studies have been widely and often mistakenly critiqued for being highly subjective, slow, costly, and of low scientific value (Flyvbjerg, 2006; Stake, 1995). The decision to use this method was therefore carefully deliberated, as recommended by Stake (1995), and the choice to pursue it was driven by the following reasons.

Firstly, single-case studies are often used to explore complex issues with limited prior research and to promote a holistic understanding of a phenomenon (Flyvbjerg, 2006; Kumar, 2011; Stake, 1995; Verschuren, 2003), making this approach well-suited to this paper’s research aim. To develop a process-based understanding of resilience, a method capable of capturing depth and richness was required—something uniquely offered by the single-case study (Westers & Peters, 2004). Furthermore, by employing this approach, this study could conduct a deep, detailed exploration of an individual experience, maximizing what could be learned from a single case while critically reflecting on broader generalizations and future research directions in resilience. That is to say, despite the emphasis on particularization, this paper’s case study is an “instrumental case study” (Stake, 1995, p. 3). It focuses on the particular in order to reflect upon issues extending beyond the case itself, aligning seamlessly with this paper’s aim.

The unique combination with IPA also strengthened the choice for a single-case study approach. While case studies are often characterized by open-ended techniques of data collection and analysis (Kumar, 2011), IPA provides concrete guidelines for gathering and interpreting qualitative data (Smith et al., 2022). Moreover, IPA explicitly focuses specifically on individuals’ interpretations of their experiences and is particularly concerned with instances when everyday experiences take on significant meaning, typically following major life events (Smith et al., 2022). As such, it offers appropriate tools for this paper to obtain a rich account of how one experiences navigating adversities associated with spousal loss. Additionally, Stake (1995) explains how when using a case study, the researcher has an ethical obligation to minimize misinterpretation and misunderstanding of the case study. IPA provides a solid framework to guide this process, strengthening integrity and robustness substantially.

Our choice for an IPA case study of one individual was therefore driven by our research aim. The single IPA case study offered the tools to best approach the task at hand. The emphasis on a single case study in this research should therefore not be regarded as a dismissal of studies that utilize larger samples or populations. Rather, it is intended to be complementary. While larger samples offer significant advantages in terms of breadth, they often suffer from a lack of depth. Conversely, case studies provide in-depth insights but lack breadth. Therefore, both approaches are essential for optimal, well-balanced and comprehensive development of research (Flyvbjerg, 2006; Verschuren, 2003).

### Participant selection

This paper is part of a larger, ongoing research project conducted in collaboration with social welfare and healthcare organizations in three Dutch municipalities. Professionals from these partner organizations acted as gatekeepers in the search for eligible participants.

Case studies and IPA conventionally work with purposively selected participants who can provide insight into a specific perspective on a phenomenon (Flyvbjerg, 2006; Smith et al., 2022; Stake, 1995). We therefore purposively sought participants aged 65 or older, comprising two men and two women, all residing in the same area of the Netherlands, whose long-term perspective on spousal loss could offer insights into the process of resilience over time. Further inclusion criteria required participants to have directly experienced spousal loss at least one year prior to the interview and to retain an active memory of the event. Exclusion criteria included mental health issues or physical limitations at the time of recruitment that would hinder safe participation.

One of the four participants found, Bas (pseudonym), was selected as the case study for this paper. The absence of a maximum time frame for partner loss made Bas’s case unique and valuable to analyse as a single-case study. Bas, a 76-year-old Dutch male, lost his wife, Elly (also a pseudonym), 30 years before the interviews. His experience, spanning three decades, provided a detailed account of changes in navigating adversity and resilience from bereavement into widowhood.

Before any interview took place with Bas, he was informed about the research project and privacy protocol in an initial briefing. Bas was also provided with a formal invitation letter, which outlined the project’s overarching aim, what he could expect from the interview, and data management. The letter also informed him that he could withdraw from the research at any time without providing a reason, and that all data from his interviews would be deleted or destroyed upon withdrawal. Bas was given “thinking time” after this meeting. Once he agreed to participate, he signed informed consent forms and only after this point did the interviews begin.

### Data gathering

Bas was interviewed four times in semi-structured interviews following the IPA guidelines by Smith et al. (2022). It was estimated that four interviews would be needed to allow for a gradual pace and to build rapport between Bas and the researcher. This was deemed necessary given the emotional nature of his story. Bas chose a location for the interviews where he felt comfortable and where interruptions were minimal: his own home. A topic list with suggested questions and probes was used to structure the interviews. All interviews were recorded with an audio recorder and ranged in duration from 55 min to 69 min. The audio files were uploaded to a secure drive at the University of Humanistic Studies in Utrecht, the Netherlands, immediately after the interviews and then deleted from the recorder. In between each interview, the interviewer would listen to the audio recording of the previous interview. Adjustments were made to the topic list questions and probes where additional information or clarification was needed. This process, from recruiting to completing the interviews, took place over 5 months.

### Data analysis

After the interviews took place, they were transcribed verbatim as required for IPA. These transcripts were also uploaded to the same secure drive as the audio files. The transcripts were then anonymized and analysed following IPA guidelines (Smith et al., 2022). Analysis therefore followed an iterative and inductive cycle, ensuring findings are firmly rooted in the interview data and that new information and perspectives were accommodated as the analysis progressed. This process involved what is called a double hermeneutic as the analyst tries to make sense of how a participant is making sense of what has happened to them. Analysis therefore began with careful reading and re-reading the interviews. This was followed by line-by-line engagement with the transcripts, where extensive notes were written on the descriptive, linguistic and conceptual aspects of the participant narratives. From these notes, experiential statements were constructed. They are “experiential” as they relate directly to the participants’ experiences and “statements” as they are constructed into statement form. This step concisely summarizes chunks of texts and extensive notes into statements, breaking up the narrative flow of the interviews.

The following step involved identification of emerging themes amongst the experiential statements. These are called Personal Experiential Themes (PET’s). To form PET’s, included experiential statements were clustered in terms of alignment with one another, altering the chronological order of the statements. Each cluster was given a title to describe its characteristics and hereby became a PET. Under each PET, experiential statements were divided further into sub-themes.

### Rigor & presentation of data

Making sense of the data requires a strict interpretative engagement on part of the researcher with a high level of reflexivity (Smith et al., 2022). To strengthen validity, extensive meetings took place between all authors to discuss all interpretations and decisions made. Any disagreements were discussed and together plans were made to adjust the analysis accordingly. The same approach was taken in the writing of this article.

Furthermore, criteria for reporting qualitative research (Jars-Qual), published by Levitt et al. (2018) were closely followed, as recommended by Smith et al. (2022). This provides guidelines on writing up qualitative research that can be operationalized well in IPA, enabling the reader to make an accurate estimate of its validity. Criteria included are, for example, clear focus on experience and/or participants sense-making, clear account of the purposive sampling in participant selection, clear indication of degree of structure in data collection, an account of how PET’s and subthemes were generated, an assessment and comment upon adequacy of data in terms of research question and presentation of findings in figures, tables and dialogue with data.

## Findings

The resulting eight PETs and sub-themes are presented in Table I. It is beyond the scope of this article to include the extensive experiential statements under each subtheme. This data is available upon request. Table II demonstrates how we for the purpose of analysis clustered the PETs under Bereavement and Widowhood. Based on these tables, findings are structured in a narrative form using quotes from the interview transcripts to provide further insight into each theme. This is in line with the recommendations of Flyvbjerg (2006) which emphasize how good case studies should be read as narratives in their entirety. Ellipses in square brackets […] indicate parts of quotes omitted, removing non-essential information for the point being made. Words enclosed within square brackets are added for clarification in quotes that may otherwise be unclear.

**Table I.** PETs and sub-themes.

PET	Sub-themes
Loved one’s behaviour during sudden serious illness is valued	Elly fell ill and died quickly afterwards Elly thought things through in detail about after her death Elly did not cry in front of others, Bas cried when alone Elly’s illness and death was like a film It was sometimes nice at the hospital
Impact of loved one dying is multifaceted	Had never experienced someone dying before Did not touch her body after she died Cremation was a memorable day No regrets from the time that Elly was in hospital or her cremation Initially difficult period when Elly died Had a period where he drank alcohol, and felt sorrow and anger Had to come to senses, for child Conversation with a pastor after Elly’s death helped Life event of losing Elly was impactful in negative and positive way There are sad sides to this life event There are positive sides to this life event Thought Elly was coming back during an earthquake Kept busy after death of Elly Life goes on whether you want to or not Was sometimes pissed off Protective of Elly’s things Work colleagues helped during loss of Elly Support of neighbours was nice
Deceased partner is still present in life	Loves Elly more and more and enjoys talking about her There are many things about Elly that made her impressive Had fun with Elly, they were both social and trusted each other Elly had bad things too Others are still positive about Elly Elly’s advice on worrying continues to offer support Knew Elly a long time Elly is prominently present but not a burden to current partner His feelings about Elly will never change Elly’s presence is non-spiritual Daily thoughts of Elly Anniversary of Elly is an important date Others remember Elly too
Deceased partner has influence on relationships after death	Had new relationships soon and later after death of Elly New partners knew/know what Elly means to him Can talk with current partner about Elly Will not remarry and future partners are not child’s mother Likes having a partner Has it good with current partner Grew closer to child after loss of Elly Writes gladly, like the poem about women in his life as sun’s
The grave is an important place	Goes to grave in difficult times and in good times Takes care of the grave The grave does not carry same significance for child Elly’s grave as a second place in the world, next to home Does various things at the grave Puts candles on the grave, Elly was afraid for the dark Will be buried there also when dies
You can build your own faith	Anger at a god he does not believe in It is not rational or hard but gives a good feeling Conversation with pastor about own faith
Becoming more emotional with age	More emotional than before Worries about grandchildren and child Missing loved ones in hard times
Edges and waves of loss are ongoing	The sharp edges are off but occur occasionally When feeling a sharp edge or wave, asks “Why?”

**Table II.** PETs across bereavement and widowhood.

Phase	PET
Bereavement	Loved one’s behaviour during sudden serious illness was valued
	Impact of loved one dying is multifaceted
Widowhood	Deceased partner is still present in life Deceased partner has influence on relationships after their death
	The grave is an important place
	You can build your own faith
	Becoming more emotional with age
	Edges and waves of loss are ongoing

## Bereavement

### Loved one’s behaviour during sudden serious death is valued

Elly’s death was sudden. One day she experienced pain, and the next day, she was admitted to hospital where she died shortly after. Bas remembers this loss vividly. It went faster than expected when Elly got sick. He couldn’t wrap his mind around it. He says it was like a film. He describes this by explaining how, you think the situation would knock you down, you imagine that you would faint, but that does not happen:

You leave in the car [after hearing prognosis], and then you think, that’s not true, it’s not true at all, soon it will all be over. Then you think, I’m dreaming now, and I’ll soon wake up from a very bad dream.

You go into a kind of autopilot, you eat, sleep and get up, there is a lot to arrange. If he hears of similar things happening to others now, it is as if this never happened to him really. That is for him, the film effect.

Elly did not cry in front of him or family. Bas did cry when he was by himself, in his car or at home in bed but not when with Elly:

I thought to myself, I cannot be the one who […] panics, or becomes very sad. I was sad, but only when I was alone […] When I was lying in bed, then I thought, how can all this be happening, what in the world is going on?

Bas describes how, during this period, he greatly admired Elly for how she approached the situation. She for example spent a lot of time clarifying how things should be after her death and gave advice. She told Bas to have flowers in the house, and to put up the Christmas tree after her death. She told him to go on holidays, buy nice wedding clothes for their child, Robin (pseudonym) if they would marry and live long and happily. She told Robin and other family members not to judge Bas if he found a new relationship quickly after her death, that she doesn’t mind. Her final words influenced him greatly then and they still do: “All those little things, down to the tiny details, you wouldn’t want to know. She was so good, you know, wow!” He recalls additional positive memories from this time in the hospital:

Then, at some point when the sun was shining again, Elly would be sitting up in bed, having made herself up beautifully, alone in the room […] we would drink coffee, and my sister was there, and my brother-in-law […] would also come by in the morning, and then we would just have a nice chat, all about trivial things, not at all about her dying.

Bas furthermore recalls moments where health care staff supported him and Elly. For example, a nurse cut and styled Elly’s hair voluntarily as Elly liked to have her hair looking nice. Also, a brother sang a song one day for Elly, which Bas, Elly, and workers in the hospital really enjoyed. He says that with such moments you could nearly forget the reality of the situation you are in. He did however find himself wondering how he could feel happiness, when “all would be gone soon”. It was a double feeling: “I was happy and thought, how is that possible? Everything is so fine and so beautiful, and soon it will all be gone! Everything …” His admiration for Elly and how she handled the situation nevertheless prevented him from panicking: “Actually, her attitude, the way she was, made me think that I couldn’t, and shouldn’t, play the pitiful boy to the outside world.”

### Impact of loved one dying is multifaceted

For Bas, death was unknown terrain. He described how this was his first time seeing a dead person and it was his wife. When she died, there were three people in the room with her. Bas, their child Robin, and a nurse. For Bas, this was a profound experience. When Elly stopped breathing, he asked the nurse if Elly was dead now and her response helped him in this moment:

She [the nurse] hardly said anything, and when she really noticed that things were going wrong, she didn’t say anything at all. Then I asked, is she dead? She didn’t say yes, she didn’t say no, she just smiled […] that was enough for me, like, ah, it’s okay now. I thought that was very admirable of her.

Soon after Elly died, Bas and Robin left the hospital room and were received by nurses who cared for them while family members made their way to the hospital. Shortly after this, he returned alone to the room where Elly was. Family recommended him to do so, as it would be his last chance before she was moved. It was striking for him how Elly looked so different and how the room looked so bare. “It was over, all of a sudden”. The care staff had put make-up on Elly after she died, she looked different. The cards Elly had received that once filled the room had been removed:

She was just pale, and they had put on lipstick, [and] something on her eyes […] and then, she looked different […] they had obviously tried their best, but when I was sitting alone with her there in the bare, it was a somewhat bare room, because it wasn’t her room anymore, then it was suddenly over, I found that, really, just unpleasant.

After a while of being silent, he spoke with Elly about how she was now “really gone”. He just stroked her hair, not her body. He was afraid she would feel cold, and he wanted to remember her as warm.

At Elly’s cremation ceremony, Bas felt that Elly was there again. She had “beautiful” makeup on, there were flowers, and the speeches of family and friends made a great impression on him:

When I, for example, saw her again later, after they had laid her in a coffin, and she was beautifully made up and all, then she was there again, yes, I appreciated that. Of course, you don’t want to see your wife in a coffin, not at all, but then […] it was Elly again. And with all the flowers around her, it started to resemble her a bit more, which was nice, actually quite beautiful, more beautiful.

All the speeches gave similar accounts of Elly. People were positive about her personality and her trademark coffee and cigarettes. They described her as positive, beautiful and happy during the speeches. The stories also contributed to the feeling that Elly was there again. Bas also gave a speech at the cremation. He had begun writing it while Elly was in the hospital. It was strange and emotional to write, seeing as she was not yet dead. It was important to him, however. He wanted to make clear what Elly meant to him. He says it was of “absolute importance” that others knew this and that she deserved a speech. She did a lot for him and now he wanted to do this for her. This helped him.

Looking back, Bas has no regrets in regard to Elly’s time in the hospital and later, her cremation, “It all fitted with who she was”.

## Bereavement into widowhood - shortly after Elly’s death

### Impact of loved one dying is multifaceted

When Elly died, Bas initially went through “a difficult time”. He panicked for a while. He did not feel himself; he pitied himself and thought about how he would have to “do it all alone”: “I am generally quite calm, and I know, also quite well, how to talk openly about things. But then, I just couldn’t see a way forward.” He says it went ok until he brought Robin back to their student residents. He did not want to go home alone, then it would become definitive he thought: “Then I honestly said, I don’t want to go home yet, I need to be here for a while, because, well, once I go home, then it’s really over.” The house had become quiet, and her death was “becoming real”. He found life hard at the beginning, “life was broken” he says: “At a certain point, everything was dark, and the sun had gone. I also couldn’t see things clearly anymore. […] Then it’s not a film. Suddenly, it’s reality.”

In the first 2–3 weeks after Elly died, Bas drank alcohol excessively and was filled with sorrow and anger. He could neither recognize beauty nor comfort in his and Elly’s beloved house:

Then I looked around and [thought] is this supposed to be the beautiful, nice house […], I didn’t like it anymore. I’m here, warm and dry, that was the only thing I found important […], nothing else mattered to me anymore.

He would listen to the cremation music and call Elly’s name out loud in an expression of frustration. He was angry, and threatened a God he did not believe in. The anger did not take over his life, he says that these moments were more about expressing anger about what could have been, and for all the memories he shared with Elly. He kept these moments of expressing anger to himself.

Drinking alcohol made him sadder and emotional he noticed, “so he stopped”. He knew it could destroy everything and that he had to watch out for Robin. He thinks sometimes that if he had no child, and if someone had offered him a pill to end his life at that time, that he would have considered it in the hope that he might see Elly again. Such thoughts were not usual for him and frightened him. He realized if Elly knew what he was doing and thinking, it would not be okay: “She would not have wanted me! Or she would just say, when I approach Peter at the gate ‘You should not let that one in! because he doesn’t belong here!’ Haha” This was not the way he had promised to look after Robin. He understood that he had to “come to his senses”.

After this initial “difficult period”, Bas kept busy. Distraction was important for him. He kept himself occupied with minding the house and garden as he had promised Elly to do so, and he was also busy at work. He had returned to work quickly after Elly’s death and emphasizes that this was not hard because his colleagues were so understanding and flexible:

Suddenly, Elly would come to mind, and I would take a break, for a moment, and then I would think, I must go to her [grave]. Then I said to the guys at the office […] “I’m going to see Elly for a bit”, and they understood that […], they knew I just needed that moment. And then I would come back a quarter of an hour, half an hour later, and then everything was okay again.

He furthermore was frustrated that he was alone in the weekends. He went looking for activities that interested him. This way he would not have to sit at home alone.

Bas also continued to attend social activities even though this was difficult. People told him if he removed himself from such things that he would “fall into a deep hole”. It was hard, and some days he was angry and unfriendly towards other people. He “weighed up” the words of others, and if they didn’t sit right with him, he could get irritated and angry: “Then every word is weighed on a golden scale, and then, if it falls wrong, it really falls wrong, while that is not like me at all!” The next day he would make up with people he was angry or irritated with. Bas emphasized how he did not always have to do this with those who knew him best: “Someone who knew me well, they knew to just let me be for a moment.”

On the other hand, Bas still appreciated support from others. He received letters, including one from a neighbour that was “a kind of poem” about him and Elly. He found this to be very beautiful. Furthermore, a card from the local butcher on the one-year anniversary of Elly’s death also made a great impression on him:

From that man, and his wife, on the one-year anniversary of Elly’s death, I received a card saying “we are thinking of you” […] and I thought, he is just a client of mine! […], yes, I had been inside [his shop] for business, but we never had a drink together or something, you know?

Since then, they send Christmas cards to one another each year. The butcher himself died a few months before the interviews, but Bas has promised to keep up the tradition with the wife of the butcher: “I sent his wife a card, and I said to her, ‘Your card made such an impression on me before, we are going to keep this up actually, until one of us is here no more.’”

He ultimately began to realize that life goes on whether you like it or not: “Life must go on, so [adapting] is necessary. Whether you want to or not, you have to do it because otherwise, you’ll ruin your own life and your surroundings too.” It was particularly important for him to account for his and Elly’s child.

Initially, he was also protective of Elly’s possessions, he did not want to “lose more of her”, but this too changed over time. He gave away most of her things to a good cause. He noticed how different items carried different meaning overtime:

Her shoes, they were here for a long time, under the chair. That was her chair, she would sometimes sit on it. […] until one time, my brother-in-law came by [months after Elly’s death], he sat on that chair, and after that, it was over with that chair. It wasn’t “that” chair anymore.

## Later in widowhood—present situation

### Deceased partner is still present in life

Elly comes to Bas’s thoughts daily. He clarifies how he does not dwell too long on this even though it happens every day. Furthermore, when Elly was alive, she would give Bas advice on worrying for example and her advice still lives with him.

I have a problem, I’m lying in bed at night, I can’t sleep, then she would say, just go to sleep, because you can’t solve that tonight in bed, you have to do it another day […], that always stays with me.

Additionally, the anniversary of Elly’s cremation remains an important date each year. He thinks more about her around this date than usual and watches the recording of the cremation ceremony:

At half past one, I put on the CD of the service that was held when Elly was cremated, at the crematorium […] I’ve been doing this for 30 years, and, for 30 years, yes, I cry about it […] just for a moment. But it’s quite possible that an hour later I’m back working in the garden, or I go shopping, and then I’ve completely forgotten about it […] I prefer to do this [watch the ceremony] alone and Robin normally comes with me to the graveyard on that day.

Bas describes how he would find it “terrible” if no one ever spoke of Elly anymore: “That’s what I always say, as long as we still talk about you, then you’re not dead, or you’re not gone […], and I find that beautiful.” His first grandchild was also named after Elly, and he enjoys this. Furthermore, people still mention Elly to him which makes him “feel good”. They sometimes send him old photos as they “know that is important to him”: “Then I think, still, she is not forgotten, and wow, they also know how important she was […] they all know how important Elly was to me”.

### Deceased partner has influence on relationships after their death

Elly has been influential in the intimate relationships Bas has had since her death, as well as in his current relationship. Firstly, her words on how he should not feel guilt when finding “someone new”, helped him deal with the double feelings of entering relationships with other women. He would remember her words when questioning himself on how he could be so happy with another. Secondly, these women understood and understand that Elly is important to him. He could and can talk with them about Elly and they all knew and know that he regularly visits her grave. Additionally, Bas decided not to remarry. For him, Elly will always be his only wife that no one can replace. He is adamant that it is neither possible nor fair to try find Elly back in a new partner: “Every person is different […], you shouldn’t compare, that’s not fair, you shouldn’t do that.” He is sure his partner understands this choice:

We are not going to get married […] that’s the only thing that distinguishes our relationship now from what I had with Elly, and what she had with her [late] husband […] It’s more out of tribute, respect, […] for the ones who are no longer here.

Bas has furthermore become closer to Robin. Elly was Robin’s best friend he says. After Elly died the bond between him and Robin changed. Together they “found fun and enjoyment in life again”:

Robin is my child, but also my friend. The one I tell everything to, almost everything, yes, really everything that I’m dealing with […] and sometimes we completely disagree, but well, haha, that’s part of it! It has to be, then we have great debates together […], I find a discussion very nice.

He emphasizes that Robin and his partners after Elly brought “light” into his life.

### The grave is an important place

Bas visits Elly’s grave every week. In especially difficult times, he visits and tells her about his problems. This gives him relief. He visited there for example when questioning how he could be happy in new relationships. He also visits in good times, to thank Elly for helping when things go well, for example, when the grandchildren do well in exams and to “share the good times” with her. He can then feel happy, but also sad that she is missing out. The grave carries great significance for him, he experiences what he describes as a feeling of “safety” and “home” there:

I feel very much at home there, it’s so familiar, it’s as if there are neighbours there, because people pass by a lot, always the same ones of course […], one time you have a chat about the weather, another time it’s about how [a decoration on a grave] looks beautiful […] there’s always something, and now again conversations like, ‘Elly [died] 30 years ago? I can remember hearing that she had passed away.

Elly was afraid of the dark, so Bas likes to sometimes place candles that burn for a long time on her grave, particularly on special occasions: “And then, I think, she does have a bit of light … yes, strange, but I find it nice.”

At times, Bas finds himself to be “childish” in how he feels so good at her grave. He recognizes himself as childish in certain actions he took and takes, like for example when he sat at the grave plot before Elly was buried there for comfort, and when he looks for signs from Elly at the grave like a bird who comes nearby. Nevertheless, he likes it there and tells Elly he will visit for as long as he can. When he dies, he will be buried there and will have “Together again” engraved on the headstone. He has agreed on this plan with his current partner. He says she understands this, and she also wishes to be buried with her late husband.

### You can build your own faith

While Bas admits feeling Elly’s presence in his life and spends time talking with Elly at her grave, he is insistent that the feeling of her presence is “not spiritual”. It is complicated, as he sometimes thinks Elly can see him, Robin and the grandchildren but he rationally believes she “probably cannot”. He thanks her for helping with different things in life, while simultaneously not believing that she is really there:

If you do not believe, then, she cannot help you, there simply lies ash [in her grave], just like from a cigarette or from an open hearth […], well, [she helps] in my head indeed, in my heart too, and in my thoughts, and I find that nice.

This feeling is similar to when Elly died, he was angry at a God he does not believe in. He says, “when it suits him”, he can look upwards to the heavens and ask ‘Why?” He cannot rationally explain these actions but does not believe he needs to “make it hard”. These actions give him a good feeling. A conversation with a pastor helped him with this:

[The pastor] said “it’s not crazy, you can believe however you want”, that’s why I keep saying: my faith is now in the graveyard […] [The pastor] was very religious, very, but he says, “no, if you find that nice, you should just do it, you shouldn’t change that, you shouldn’t change because it’s necessary, it’s not necessary.”

### Becoming more emotional with age

Bas highlighted in the interviews that he is also becoming more emotional as he grows older, and he is “more often sad” than when he was younger. He finds himself worrying more than before about something happening to Robin or the grandchildren. He also notices especially that in harder times he misses Elly more than before:

Or sometimes when I feel down, I think, damn it, I wish Elly was still here! Or that I could see her one more time. But that comes with getting older, I think. I have the same about my mother.

### Edges and waves of loss are ongoing

For Bas, the “sharp edges” of the pain around what happened are mainly gone. He says it is like the waves of sadness begin deep and low. Over time the waves flatten, and Elly’s death feels far away. Then all of a sudden, a wave can come again, and he can suddenly feel angry and sad about why it all happened. New waves of grief are sometimes triggered when he sees women of her age for example, and he wonders what could have been. Special moments can also cause such waves, when thinking of what she is missing out on, such as Robin’s wedding or the birth of grandchildren. The waves now come rarely however, and he feels happier about his time with Elly than sad about why it had to end.

Bas spoke positively about Elly throughout the interviews and emphasized at the end that he hopes it comes across how much he loved her:

There’s hardly a day that I don’t think of her for a moment. It’s not that it necessarily has to happen, but it just comes all at once … boom, then she’s there […] From my life, she will never be gone, even though she’s no longer here.

## Discussion

This paper offers a uniquely rich and nuanced perspective on the dynamic nature of resilience as it unfolds over time. Tracing Bas’s experience of spousal loss over thirty years deepens knowledge and understanding of the three essential elements of an operational definition of resilience: adversity, outcomes, and mechanisms. Moreover, the findings illustrate how these elements interact and are experienced over time, offering valuable insights into resilience from a process-based perspective.

Firstly, findings highlight how *adversity* plays a key role in shaping resilience as emphasized in Glas et al. (2023). This case study illustrates how adversity assumes a dynamic mix of forms. This confirms how, for example, while adversity manifests in extraordinary events themselves, it is also present in everyday, seemingly mundane activities as highlighted in Wright St-Clair et al. (2011). Take for purpose of illustration the double feeling Bas experienced when having “positive moments” in the hospital while Elly was dying, deciding not to touch her body after she died, coming home to an empty house after her death, uncomfortably attending social events and not being able to focus on work. Or take examples of adversities he faced and is facing later in widowhood in feelings of guilt upon entering new relationships, unexpected waves of grief, experiencing more sadness and worry in older age. As emphasized in Power et al. (2019), these challenging situations come with pain, disruption, exhaustion, disorientation, loneliness, and grief to varying degrees, influencing Bas’s responses and resulting outcomes. What supports Bas in navigating each of these adversities is different from situation to situation, which stresses how each experience of adversity, ordinary and extraordinary, is embodied and embedded in different contexts.

Secondly, regarding *outcomes*, findings align with Lenette et al. (2013) in how Bas experienced a complex and fluctuating mix of several “more positive” and “more negative” outcomes that occur in navigating the diverse adversities he faces. This case highlights how like adversity, these outcomes can play a pivotal role in the resilience process and are not “end-stations”. What can be identified as positive outcomes in Bas’s story, seemingly bear precursors for potential negative outcomes and negative outcomes in turn, bear precursors for potentially positive ones. For example, Bas initially experienced what he called a “difficult time”. He was struggling and panicking. He drank alcohol excessively and experienced frightening thoughts. Findings illustrate, however, how this “difficult time” was infused with the precursors for recovery and growth. What can be labelled as adverse outcomes in the face of spousal loss, namely excessive alcohol use and frightening thoughts, became the new adversities that confronted him daily. Becoming sadder and seeing that he “could destroy everything” if he continued this way, helped shift his focus, which stimulated the first steps of recovery, transformation and growth. He went back to work, attended social activities, became closer to his and Elly’s child, kept busy and entered new relationships. The findings of this research however again emphasize that in these positive outcomes, there is always a subtle permeation of enduring pain and loneliness surrounding Elly’s death. Some days this is less subtle than others. This is true even now, thirty years after Elly’s death when he is faced with a sudden wave of anger and sadness.

Correspondingly, these precursors remain latent or manifest, contingent upon the third essential element *mechanisms.* These are the interactions that facilitate the various outcomes when facing adversity. Findings underscore how various resilience enhancing resources, or factors, on different levels are key to these mechanisms, as written in Angevaare et al. (2020). On an individual level for example, Bas shows personal capacity for openness and honesty about a continued bond with Elly. This was expressed in new relationships with other women, and in conversations with pastors for example. On a relational level, the findings illuminate an evolving web of social interactions that supported Bas over time. To name some examples, take the sensitive care of healthcare staff, the supportive colleagues, the butcher’s card initiating a thirty year long connection, the network of people Bas has come to know at the graveyard, the encouragement to attend social events, the spiritual guidance provided by pastors, the development of a closer relationship with his child as well as understanding and subsequent support in new intimate relationships.

Findings from this study provide a novel account of how Bas experienced enacting such resilience enhancing factors and resources. Take, for example the inspiration Bas draws from his bond with Elly. This supported him in the lead up and aftermath of her death. While this can be seen as a resilience strengthening factor for Bas, resulting in positive outcomes, it was and is nevertheless challenging for him to enact this in his daily life. He for example visited her grave, feeling confused about entering new relationships even though he was inspired by her final words when she told him she would not mind. Furthermore, while her advice on worrying continues to inspire him in especially hard times, it also simultaneously makes him miss her, and wish she could be here. For purpose of further illustration, take how important network is to Bas as a resilience strengthening factor. His colleagues helped him return to work with their flexibility and it does him good when people talk of Elly or send him photos and cards. However, contact with others was also simultaneously difficult for Bas at times. Attending social events was difficult, and he was very sensitive to the words of others. While we are aware of resilience strengthening factors, Bas’s story illustrates how implementing them to lead to positive outcome is not always easy and in fact forms an uncertain, contradictory and bumpy road.

A temporal dimension of the resilience process (Fisher et al., 2019) is also strikingly evident in the findings. For example, the dynamic nature of adversity, outcomes, resilience-enhancing factors, and mechanisms over time can be traced by examining distinct life events or phases in Bas’s story. These include the immediate crisis period at the hospital, Elly’s death, the transition period directly after her death, widowhood, and growing older. Consider for instance various meaning-making practices and rituals involved in these phases—some evolve over time, while others remain constant. Examples include calling Elly’s name out loud in the weeks after her death, an evolving connection with physical objects (her chair and shoes), visiting the grave weekly for thirty years, and watching the cremation service annually on the anniversary of her cremation. These life events or phases function as useful temporal markers, enabling the mapping of resilience mechanisms from a process-based perspective over a thirty-year period.

The findings in this paper ultimately offer a rare and richly illustrated account of resilience as a dynamic and evolving process. By tracing Bas’s experience over three decades—from young widower to grandparent, from immediate crisis to long-term adaptation, and from raw grief to the integration of loss—this study highlights how Bas navigated challenges, stabilized, learned, and developed sustainable practices and rituals, continuously adapting to new circumstances. This trajectory closely aligns with Bourbeau’s (2018) concept of “bouncing forward,” emphasizing resilience as an ongoing process of transformation and renewal rather than a return to a pre-adversity state. This study also illustrates how identifying key temporal markers (life events or phases) offers a systematic framework for analysing this dynamic process. Additionally, a continued supportive bond with Elly after her death is evident in how Bas emphasizes that Elly will “never be gone from his life, even though she is no longer here” and how he continues to draw inspiration and comfort from this. This demonstrates how Bas’s connection with Elly endures over time, reinforcing the theory of continued bonds (Klass et al., 1996), and contemporary models of grief (Stroebe & Schut, 2010; Worden, 2008). Finally, while not the focus of this paper, this study provides a rare, idiographic perspective on male widowhood, an area that remains underrepresented in grief and resilience research (Ang, 2023; Bennett et al., 2003; Naef et al., 2013; Rodger et al., 2006; Yu et al., 2021). While this study does not explicitly apply a gender analysis, it offers insights that could inform future research on the intersection of gender and grief.

## Limitations and strengths

IPA employs a “double hermeneutic,” in which the researcher actively interprets the participants’ own interpretations of their experiences (Smith et al., 2022). Given this interpretative process, it is essential for IPA publications to reflect on the positionality of the lead researcher and how this has shaped the research process, data interpretation, and findings.

Firstly, the lead researcher’s background in social care and qualitative research provided expertise and interpersonal skills that enhanced the data collection process. Her training in fostering trust and openness advanced her ability to establish rapport with Bas. This, combined with her background in qualitative research, provided experience in framing follow-up questions to subtly deepen the interviews and facilitated the navigation of sensitive conversations with care and attentiveness. Nevertheless, her natural tendency to lean towards “helping” or “intervening” rather than “listening” introduced certain complexities. For instance, the utmost caution was required to prevent providing unnecessary support or influence during the interviews, especially when Bas faced difficult emotions or struggled to find words.

To mitigate these challenges, the lead researcher engaged in ongoing reflexivity and regular supervision, critically reflecting on instances where maintaining the role of researcher, rather than that of a social care professional, was particularly challenging. Additionally, listening to interview recordings between sessions helped identify moments where her social care background may have influenced the conversation, allowing for clarification with Bas and adjustments in subsequent interviews. During the interviews, particular attention was also given to allowing extended pauses and using open-ended questions, ensuring that Bas had the time and space to articulate his thoughts and lead with his narrative.

Secondly, the lead researcher has extensive experience with the topic of resilience. Preparation for this article naturally required extensive reading on the topic. Furthermore, this article forms part of a larger research project on resilience and different types of loss, utilizing action research methods involving 195 participants at the time of this publication. Such expertise and experience can strengthen both data collection and analysis by providing a theoretically and empirically informed foundation, enabling a nuanced and sophisticated approach to interpretation. However, this can also create certain limitations in how interpretative focus can become shaped. For instance, specific questions or the framing of results may place greater emphasis on particular aspects than Bas himself originally did.

To address potential bias emerging from experience and expertise on resilience, extra vigilance was applied in adhering to IPA’s iterative engagement with the transcripts. This structured approach facilitated the distinction between interpretations grounded in Bas’s own words and those reflecting the researcher’s conceptual and theoretical reflections on his interpretations. Furthermore, the extensive and critical discussions amongst all authors contributed to identification of implicit biases. This approach ensured that all interpretations remained open to counter-narratives, thereby clarifying what was directly grounded in Bas’s account and what emerged from the researcher’s interpretation, along with the rationale behind these interpretative choices.

By employing the above measures, a balance was achieved in this paper, between leveraging the strengths of the first author’s background and expertise while maintaining a commitment to giving equal weight to Bas’s interpretation of his own experience.

## Conclusion

Findings of this paper add to the current body of research on resilience in later life as it richly illustrates a particular lived experience of resilience during spousal loss from a process-based perspective. Adversities, in this case, range from the extraordinary to the mundane, and outcomes span from more positive to more negative. Findings demonstrate how the diverse adversities and various outcomes encountered can be experienced as being diffused through one another, interacting with and shaping one another over time. Mechanisms enacted to utilize various resilience-strengthening factors or resources in navigating this dynamic and sometimes contradictory process, create a challenging and bumpy path. This resembles a process of “bouncing forward” as written in Bourbeau (2018).

While Bas’s story illustrates the integration of spousal loss into life, this is not always the case, particularly in instances of chronic or complicated grief. Publishing IPA case studies on spousal loss with diverse participants can deepen understanding of the complexity of resilience from a process-based perspective, making its definition and practical applications more grounded in everyday experiences. Additionally, such studies can inform cross-case comparisons and guide larger-scale qualitative and quantitative research on resilience as a dynamic process. Based on the findings of this paper, future research should not only consider context when selecting methodologies but also focus on how individuals actively interpret and reshape their experiences over time. Methods that capture experiences of this evolving meaning-making processes—whether through IPA or complementary approaches—can offer valuable insight into resilience as a continuously unfolding process.

## Data Availability

The data package for this paper includes four anonymized interview transcripts. Given the sensitivity and depth of data inherent in a single-case study, these transcripts are securely stored on a protected drive at the University of Humanistic Studies in the Netherlands. Access to this data may be granted upon reasonable request by contacting the corresponding author, subject to ethical and confidentiality considerations.

## CRediT statement

Conceptualization: CB, AN, RB, AM

Funding acquisition: CB, AN, RB, AM ZonMw [Project number: 05550032110010]

Methodology: CB, AN, AM

Project administration: CB, AM

Investigation: CB

Supervision: AN, AM

Formal analysis: CB, AN, RB, AM

Data curation: CB, AM

Writing—original draft: CB

Writing—review and editing: CB, AN, RB, AM
